# Changes in PGC‐1α/SIRT1 Signaling Impact on Mitochondrial Homeostasis in Amyloid-Beta Peptide Toxicity Model

**DOI:** 10.3389/fphar.2020.00709

**Published:** 2020-05-21

**Authors:** Jessica D. Panes, Pamela A. Godoy, Tiare Silva-Grecchi, María T. Celis, Oscar Ramirez-Molina, Javiera Gavilan, Carola Muñoz-Montecino, Patricio A. Castro, Gustavo Moraga-Cid, Gonzalo E. Yévenes, Leonardo Guzmán, Jeffrey L. Salisbury, Eugenia Trushina, Jorge Fuentealba

**Affiliations:** ^1^Laboratory of Screening of Neuroactive Compound, Physiology Department, Faculty of Biological Sciences, Universidad de Concepción, Concepción, Chile; ^2^Physiology Department, Faculty of Biological Sciences, Universidad de Concepción, Concepción, Chile; ^3^Neurology Research, Mayo Clinic Foundation, Rochester, MN, United States; ^4^Center for Advanced Research on Biomedicine (CIAB-UdeC), Physiology Department, Faculty of Biological Sciences, Universidad de Concepción, Concepción, Chile

**Keywords:** mitochondrial dysfunction, Alzheimer's disease, amyloid beta oligomers, PGC-1α, SIRT1, DRP1, Mfn1

## Abstract

Alzheimer's disease (AD) is a neurodegenerative disorder characterized by cognitive impairment that increasingly afflicts the elderly population. Soluble oligomers (AβOs) has been implicated in AD pathogenesis: however, the molecular events underlying a role for Aβ are not well understood. We studied the effects of AβOs on mitochondrial function and on key proteins that regulate mitochondrial dynamics and biogenesis in hippocampal neurons and PC-12 cells. We find that AβOs treatment caused a reduction in total Mfn1 after a 2 h exposure (42 ± 11%); while DRP1 increased at 1 and 2 h (205 ± 22% and 198 ± 27%, respectively), correlating to changes in mitochondrial morphology. We also observed that SIRT1 levels were reduced after acute and chronic AβOs treatment (68 ± 7% and 77 ± 6%, respectively); while PGC-1α levels were reduced with the same time treatments (68 ± 8% and 67 ± 7%, respectively). Interestingly, we found that chronic treatment with AβOs increased the levels of pSIRT1 (24 h: 157 ± 18%), and we observed changes in the PGC-1α and p-SIRT1 nucleus/cytosol ratio and SIRT1-PGC-1α interaction pattern after chronic exposure to AβOs. Our data suggest that AβOs induce important changes in the level and localization of mitochondrial proteins related with the loss of mitochondrial function that are mediated by a fast and sustained SIRT1/PGC-1α complex disruption promoting a “non-return point” to an irreversible synaptic failure and neuronal network disconnection.

## Introduction

Alzheimer's disease (AD) is a type of dementia characterized as a progressive brain disorder with high prevalence in elderly people. The etiology of AD has not been completely determined. The time course of the disease involves the loss of cognitive abilities and behavioral changes related to progressive neuronal failure and death, mainly in the hippocampus and cortex (Castellani et al., 2010). There are two classical histopathological biomarkers detected in AD: neurofibrillary tangles and amyloid plaques that are intracellular aggregates of hyperphosphorylated microtubule-associated protein tau (Kosik et al., 1986) are insoluble and extracellular aggregates of the β-amyloid peptide (Aβ), respectively (Glenner and Wong, 1984). Aβ monomers aggregate forming soluble oligomers composed of 3–24 monomers, known as soluble oligomers (AβOs), these can produce alterations in dendrite spine morphology in hippocampal neurons (Shankar et al., 2007; Parodi et al., 2010). AβOs provide a unifying mechanism for initiation of AD pathogenesis. It has a preference for central nervous system (CNS) (Ferreira and Klein, 2011), especially in the synapses zones (Gong et al., 2003). AβOs could be the responsible for initiation and the main agent of chronic neurotoxic effects, rather than monomers and fibrils, that do not correspond to neuroactive forms of Aβ able to depress synaptic transmission (Parodi et al., 2010). Additionally, it was found that AβOs treatments on different scales emulated the main aspects of the disease, i.e. neuronal loss, calcium dyshomeostasis, synaptic failure, and particularly ATP leakage and P2XR overexpression (Fuentealba et al., 2011; Saez-Orellana et al., 2016).

It has been found that the consequences of Aβ peptide on mitochondrial function could promote early synaptic loss (Manczak et al., 2006; Schmitt et al., 2011). One of the main proposed mechanisms for Aβ toxicity is the formation of a non-specific pore in the cell membrane (Lal et al., 2007) that allows the passage of molecules and ions (Näslund, 2000; Parodi et al., 2010). Thus, Ca2+ influx through this pore has been suggested as one of the main causes of excitotoxicity in AD (Sepulveda et al., 2010), mitochondrial Ca^2+^ overload and dysfunction; this could be as consequence of plasma membrane pore formation or by direct interaction of AβOs to mitochondrial cristae (Manczak et al., 2006; Arrazola et al., 2017; Toglia et al., 2018), issue that are still under discussion. A correlation between progressive reduction in brain glucose metabolism and a decreased expression of either nuclear or mitochondrial genes related to oxidative phosphorylation (Chandrasekaran et al., 1996; Zolezzi et al., 2017; Terada et al., 2020) and mitochondrial size (Zhu et al., 2006) has been described on AD patients. These defects in mitochondrial dynamic proteins lead to impaired mitochondrial function in neurons (Liesa et al., 2009; Lee et al., 2012; Pham et al., 2012). The events of fusion and fission are highly coordinated by proteins like Mfn1, Mfn2 (mitofusin 1, 2), and OPA1 (mitochondrial dynamin like GTPase) involved in cristae fusion, while DRP1 (dynamin-related protein) and Fis1 (mitochondrial fission 1 protein) have been related with the constriction of mitochondrial cristae which lead to mitochondrial fission (Wang et al., 2014).

For example, the levels of OPA1, Mfn1, and Mfn2 were significantly reduced, whereas the levels of Fis1 were significantly increased in AD hippocampus (Wang et al., 2009). Defects in the fission process lead to an accumulation of mitochondria in the cell body and reduced dendritic and synaptic mitochondrial content (Li et al., 2004). Alterations in mitochondrial morphology, particularlyfragmented mitochondria, were found in fibroblasts and brain tissue from AD patients (Wang et al., 2008), by the interaction of Aβ with DRP1 (Reddy et al., 2011; Zhang et al., 2016).

It has been postulated that the loss of mitochondrial biogenesis is key to understanding how mitochondrial dysfunction in AD patients occurs (Hirai et al., 2001). This process is controlled by PGC-1α (peroxisome proliferator-activated receptor-γ coactivator-1α), a fasting-induced transcriptional coactivator recruited during PPAR stimulation (Viswakarma et al., 2010). SIRT1 (silent information regulator 2 homolog 1), a NAD+-dependent histone deacetylases (HDAC) deacetylase, can interact and deacetylate PGC-1α, and this deacetylation results in the upregulation of its transcriptional function (Lehman et al., 2000; Rodgers et al., 2005; Qin et al., 2009). SIRT1 is essential for maintaining cellular survival, cognitive function, and synaptic plasticity (Feng et al., 2009). Particularly in oxidative stress environments, SIRT1 regulates diverse processes such as apoptosis, cellular senescence, glucose homeostasis, and aging (Haigis and Sinclair, 2010). Importantly, it has been described that PGC-1α and SIRT1 require changes on its localization in response to stimuli (Anderson et al., 2008; Bai and Zhang, 2016). While the PGC1α-NRF-TFAM pathway was found to be downregulated in animal model of AD (Sheng et al., 2012) showing a decreased expression level in PGC-1α (Lehman et al., 2000), as changes on SIRT1 activity (Bonda et al., 2010; Godoy et al., 2014).

In this work, we assessed the effects of AβOs on early mitochondrial dysfunction and the impact on the main mitochondrial homeostasis controllers. We hypothesized that AβOs downregulate PGC-1α and the downstream mitochondrial biogenesis and dynamic process. To test this hypothesis, we used PC-12 cells, cultured mice hippocampal neurons, and hippocampal slices as models to study the effects of soluble oligomers of Aβ1–40 on mitochondrial biogenesis by measuring the expression of the SIRT1-PGC-1α pathway. Using cellular and molecular approaches, we evaluated the effects of AβOs on proteins involved in mitochondrial health and how that impacts neuronal and synaptic function.

## Materials and Methods

### Cell Culture

A PC-12 rat pheochromocytoma cell line was cultured in DMEM with glutamine (Dulbecco's modified Eagle medium, Invitrogen, Grand Island, NY, USA), supplemented with 5% fetal bovine serum, 5% serum horse (HyClone, South Logan, UT, USA), and 1% streptomycin-penicillin (Invitrogen, Grand Island, NY, USA). The cells were plated in 12-well plates at a density of 2.5x105 cells/ml. Cultures were maintained at 37°C with 5% CO_2_. The cells were used when 70–80% confluence was achieved. All experiments are performed in triplicate for three independent experiments.

### Primary Hippocampal Cultures

Eighteen to nineteen days pregnant C57BL/J6 mice were treated in accordance with regulations recommended by NIH (National Institute of Health, USA) and the ethics committee at the University of Concepción. Mice were deeply anesthetized by CO2 inhalation before being sacrificed by cervical dislocation. Primary cultures of embryonic hippocampi were prepared as previously published (Fuentealba et al., 2011) and plated at 320,000 cells/ml on coverslips coated with poly-L-lysine (Trevigen, Gaithersburg, MD). Cultures were maintained at 37°C with 5% CO_2_. All experiments are performed in triplicate for three independent experiments.

### Mice Brain Slices and Immunohistochemistry

Brain slices were obtained from C57BL/6J mice (animals were manipulated in accordance with the ethical regulations established by NIH and University of Concepción). Brain slices (120 µM) were obtained on a Leica VT1200S vibratome following the Káradóttir & Attwell method (Karadottir and Attwell, 2006). Once cut, the slices were incubated for 1 h in oxigenated ACSF solution (95% O2–5% CO2) at 34–37°C. The slices were transferred to a culture plate containing ACSF solution and treated with AβOs (0.5 μM) for 1 and 5 h. All steps in the immunohistochemistry protocol were done at ice-cold temperature and constant agitation. Slices were fixed with 500 µl 4% paraformaldehyde for 35 min. Once fixed, slices were washed five times with PBS (5 min per wash) and then incubated in blocking/permeabilization solution (0.3–0.5% Triton X-100 + 10% horse serum + PBS) for 1 h. Then, slices were incubated with the primary antibodies at ice cold temperature for 3 h (or overnight at 4°C). Antibodies used were PGC1α (rabbit, 1:400, Novus Biologicals, NBP1-04676) and MAP2 (mouse, 1:400, Santa Cruz, A-4) prepared in a solution containing 0.3% Triton X-100 + 10% horse serum + PBS. Subsequently, slices were washed five times for 3 min each and incubated with the corresponding secondary antibody (conjugated with either Cy3 or AlexaFluor488) for 90 min. After incubation, nuclear staining was performed with DAPI 300 nM for 15 min. Afterwards, slices were washed five times for 3 min each and mounted with DAKO fluorescent mounting media. Stack images were acquired on a LSM780 NLO Zeiss confocal microscope using a 63X/1.4NA objective (oil immersion). Images were processed using both ZEN software (Carl Zeiss MicroImaging GmbH) and Image J (NIH, USA). Immunoreactivity was quantified with ImageJ for both primary antibodies using 15 regions of interest in each slice within each stack for each experimental condition. PGC1α immunoreactivity was normalized for each stack of 15 ROIs were randomly selected.

Data was analyzed with GraphPad Prism Software (GraphPad Software, Inc.) where all statistical calculations (one way ANOVA with Kruskal-Wallis test, Dunn's multiple comparison test for stack quantification, and one way ANOVA with Dunnet's multiple comparisons test for single plane quantification) were done. All experiments a performed in triplicate for three independent experiments.

### Aβ_1–40_ Aggregation

A lyophilized stock of Aβ_1–40_ (rPeptide, Bogart, GA, USA) was reconstituted in DMSO at a concentration of 2.3 mM. 2 µl of this solution was diluted in sterile distilled water to reach a concentration of 80 µM. Subsequently, the peptide was aggregated at 500 rpm for 4 h at room temperature. Soluble Aβ oligomers (AβOs) were used to treat the cells and tissues for 1, 2, 5, and 24 h at a final concentration of 0.5 µM. The identity of oligomeric Aβ species (AβOs) were checked by silver staining and electronic microscopy (were performed as previously published (Sáez-Orellana et al., 2018)).

### Transmission Electron Microscopy

Ten microliters of Aβ_1–40_ at a concentration of 80 µM was applied to carbon-coated Formvar grids. Samples were fixed with a 2% glutaraldehyde solution for 5 min was exposure to colloidal beta amyloid antibody (MOAB-2, 1:50, 1 h) and gold particles (10 nm) conjugated with a secondary antibody (mouse, 1:500). The aggregates were stained with 5 µl of 0.2% (w/v) phosphotungstic acid (PTA) and the grid was air-dried. Samples were examined using a JEOL 1200 EX II electron microscope.

### Western Blot

To study the expression of mitochondrial biogenesis and dynamics proteins, we prepared whole cell lysates with a buffer containing 10 mM Tris, 10 mM EDTA, 100 mM NaCl, 0.5% Triton, 10% glycerol, and protease inhibitors. Culture lysates were mixed with 4X loading buffer and denatured at 95°C for 10 min. Then, samples were subjected to SDS-PAGE in 10% acrylamide gels at 100 V for 100 min and transferred to nitrocellulose membranes at 250 mA for 120 min. Membranes were blocked with 5% non-fat milk in TBS 1X-Tween for 1 h. The primary antibodies SIRT1 (mouse, 1:1,000, Novus Biologicals, 1F3), PGC-1α (rabbit, 1:500, Novus Biologicals, NBP1-04676), DRP1 (rabbit, 1:2,000, Novus Biologicals, NB110-55288), Mfn1 (rabbit, 1:1,000, NBP1-51841), and β-actin (mouse, 1:1000, Santa Cruz Biotechnology, AC-15) were incubated overnight. Anti-rabbit-HRP (1:5,000, Santa Cruz, Biotechnology, sc-2004) and anti-mouse-HRP (1:5,000 Santa Cruz, Biotechnology, sc-2005) were used as secondary antibodies and were incubated for 1 h. Immunoreactive signals were detected using the Western Lighting kit (Perkin Elmer, USA) andquantified with an Odyssey imaging system (Li-Cor, Lincoln, NE, USA).

### Co-Immunoprecipitation (Co-IP)

To evaluate the interaction between SIRT1 and PGC-1α, we prepared lysates from PC12 cells control and treated 24 h with AβOs. Protein concentration was normalized for the different samples. Then, the lysates were precleared with 10 µl of Protein A/G PLUS Agarose Immunoprecipitation Reagent (Santa Cruz Biotechnology Y, INC. sc-2003) and the resulting mixture was incubated at 4°C for 1 h. Subsequently, the precleared samples were incubated for 1 h with 1 µg of an anti-PGC-1α rabbit monoclonal antibody (Novus Biologicals, NBP1-04676) or a rabbit control IgG (Sigma-Aldrich, 12–370), per mg of protein. Finally, we added 50 µl of Protein A/G PLUS Agarose Immunoprecipitation Reagent to each sample and incubated the mixture overnight. Proteins were eluted in 2X loading buffer at 100°C for 8 min, analyzed by SDS-PAGE, and visualized by western blot using anti-PGC-1α and anti-SIRT1 antibodies (Novus Biologicals).

### Immunocytochemistry

To study Aβ_1–40_ toxic effects on mitochondrial biogenesis, dynamics proteins, organization, and distribution, PC-12 cell cultures were treated with 0.5 µM AβOs 1–40 for 1, 2, and 24 h. The cells were washed with PBS1X and fixed with 4% paraformaldehyde for 10 min at room temperature (RT). Later, cells were permeabilized with 0.1% Triton X-100 and blocked with 10% horse serum for 30 min at RT. Samples were incubated for 1 h at RT with the following primary antibodies: SIRT1 (mouse 1:300, Novus Biologicals, IF3), PGC-1α (rabbit 1:400, Novus Biologicals, NBP1-04676), Ser-46 SIRT1 (Sigma 1:200, SAB4301426), DRP1 (rabbit 1:200, Novus Biologicals, NB 110-55288), and Mfn1 (rabbit 1:200, Novus Biologicals, NBP1-51841). Subsequently, the corresponding Cy3 or Alexa Fluor 488 conjugated were incubated for 45 min. Then, samples were incubated with 300 nM DAPI nuclear staining for 10 min and mounted using DAKO immunofluorescence mounting media (Dako, Glostrup, Denmark). Images were acquired using a LSM780 NLO confocal microscope (Carl Zeiss Microscopy, Jena, Germany), a Nikon Eclipse TE2000-U epifluorescence microscope, and Zeiss SR-SIM equipment (Elyra S1 model), which was equipped with a DPSS Diode laser (488 and 561 nm of excitation). The 63X/1.4 NA oil immersion objective with an additional 1.6X lens was used which explains why the total increase was 1008X. Images were processed, and quantification was performed using Image J (NIH, Bethesda, MD, USA). Images were deconvolved considering the following parameters: immersion oil refractive index (1.518), objective numerical aperture (1.4), and wavelength (510 nm). Immunoreactivity for mitochondrial biogenesis and dynamics proteins was quantified. Additionally, nuclear and cytoplasmic immunoreactivity was analyzed using three aleatory areas in both cases and quantifying the mean intensity for each cell in three independent experiments.

### Digital Imaging Processing of Mitochondria Morphology Analyses

To study AβOs1–40 toxic effects on mitochondrial morphology, PC-12 cells and hippocampal neurons were stained with a specific primary antibody for TOM 20 (sc-17764, mouse monoclonal, F-10, outer mitochondria membrane). Images were acquired using a LSM780 NLO confocal microscope (Carl Zeiss Microscopy, Jena, Germany) or superresolution microscopy (see above). After acquisition, images were processed with Image Processing and 3D reconstructions were performed using BitPlane IMARIS 9.1 software to evaluate the shape and area of MTG-stained cells. Briefly, binary objects were obtained by image segmentation, and subsequently an automatic measurement tool was used to calculate the volume and elongation of each mitochondrion.

### Superresolution Microscopy

Images by structured illumination were obtained using a Zeiss SRSIM model Elyra S1 superresolution microscope equipped with a DPSS diode laser (561 nm excitation). An oil immersion 63x/1.4 NA objective was used with an additional 1.6x lens for a total magnification of 1008x. The emission filters used were a BP570-650 and LP750 for the fluorophores FITC and Cy3, respectively. For all captured images a configuration with five grid positions was used (28 μm for the 488 nm laser and 34 μm for the 561 laser), with a z-stack spacing of 110 nm between each plane captured. Images were processed using Image J software (NIH, Bethesda, MD, USA).

### Data Analysis

The obtained data was analyzed using GraphPad Prism 6 Software. Normality was verified using the Kolmogorov-Smirnov test or Shapiro-Wilk normality test depending on sample size. In the case of normal data, statistical significance was determined using unpaired Student's t-test (for two groups) orone-way ANOVA followed by the Dunnett's multiple comparisons test (for multiple group comparisons). In the case of non-normal data, statistical significance was determined using Mann-Whitney test (for two groups) or Kruskal-Wallis test followed by the Dunn's multiple comparisons test (for multiple group comparisons). All experimental data are expressed as the mean ± SEM (unless otherwise indicated). P-values < 0.05 were considered statistically significant. Exact n for each experiment is reported in Figure legends.

### Animal Manipulations

All procedures related with animal management and tissue isolation were done following NIH (USA) and CONICYT guidelines and with protocols approved by the bioethical committee of the Universidad de Concepcion. 297

## Results

### AβOs Induced an Imbalance in Mitochondrial Fission/Fusion Proteins in PC-12 Cells

**Figure 1:**
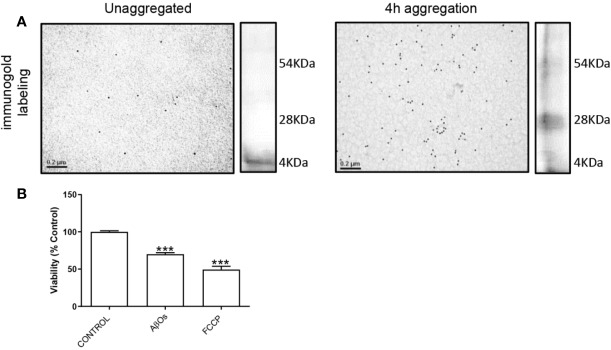
Characterization of AβOs. **(A)** Microscopy electronic of A**β**_1–40_ peptide. A**β**_1–40_ peptide was aggregated 4 h and was exposure to colloidal gold particles (10 nm) conjugated with a β amyloid Antibody (MOAB-2). A**β**_1–40_ peptide aggregation is visualized by silver staining to the right side of each panel. **(B)** PC-12 cells were incubated with AβOs 0.5 µM for 24 h and FCCP (10 µM). Data are represented as mean ± SEM. ***p < 0.001 compared between the control group. One-way ANOVA used for all statistical analyses. FCCP, Carbonyl cyanide-4-trifluoromethoxy) phenylhydrazone. (n=3–5 for each group).

To characterize the Aβ_1–40_ aggregates, we performed transmission electron microscopy and silver staining experiments to confirm the presence of Aβ_1–40_ oligomers (preparations used in this study, **Figure 1**). The data showed that the soluble oligomers of Aβ_1–40_ were principally low molecular weight structures (**Figure 1A**). Moreover, these AβOs (**Figure 1B**) displayed similar than toxic effects mitochondrial uncoupler FCCP (10 µM). With these experimental approaches, we confirm that the Aβ species used in this were mainly toxic oligomers. We decided the use of Aβ_1–40_ instead of Aβ_1–42_, because Aβ_1–42,_ aggregation process is difficult to control which usually promote the formation of less active aggregates (Pauwels et al., 2012). Furthermore, we have characterized β-amyloid effects of different Aβ species (monomers, soluble oligomers, or protofibrils), and we observed that monomeric species of Aβ have a neurotrophic-like effects (Cuevas et al., 2011); while soluble oligomeric species of Aβ effects were compared with insoluble fibrillar species (Parodi et al., 2010; Sepulveda et al., 2010); taken together, we corroborated that the soluble oligomers, have the active toxicity related to synaptic failure described by our group previously.

**Figure 2:**
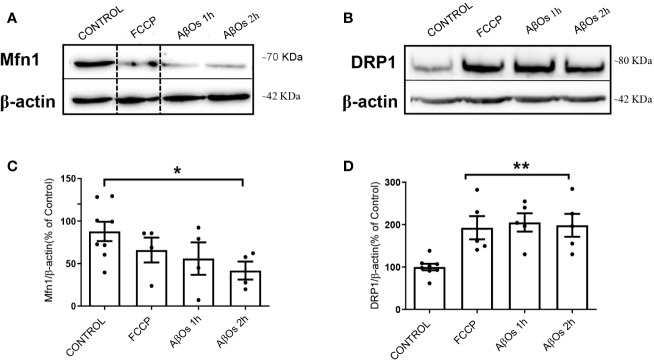
Acute effects of AβOs on total levels of proteins that regulate mitochondrial dynamics. **(A)** Mfn1 and **(B)** DRP1 western blot of lysates from PC12 cells treated with FCCP (10 μM, 2 h) and AβOs (0.5 μM, 1 and 2 h). A dotted line on the western blot indicates different regions of the same gel. **(C, D)** Quantification of Mfn1 and DRP1 levels normalized to beta actin. Data are represented as mean ± SEM. *p < 0.05, **p < 0.01 compared between the control group. One-way ANOVA with the Dunnett's multiple comparisons test was used for all statistical analyses. Mfn1, mitofusin 1; DRP1, dynamin-related protein 1. (n = 4–5 for each group) (original gel blot are provided on **Supplementary Figure 2**).

**Figure 3:**
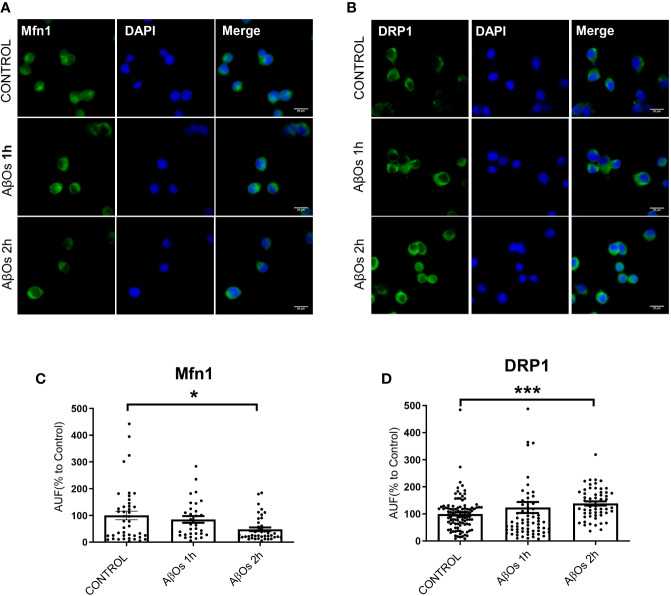
Acute effects of AβOs on immunoreactivity of proteins that regulate mitochondrial dynamics. Representative epifluorescence images of **(A)** Mfn1 and **(B)** DRP1 immunoreactivity in PC-12 cells control and treated with AβOs (0.5 μM) for 1 and 2 h. Quantification of **(C)** Mfn1 and **(D)** DRP1 immunoreactivity (intensity), under the same experimental conditions. Scale bars: 20 μm. Data are represented as mean ± SEM. *p < 0.05, ***p < 0.001 compared between the control group. One-way ANOVA with the Dunn's multiple comparisons test was used for all statistical analyses. Mfn1, mitofusin 1; DRP1, dynamin-related protein 1. (n=3–6 for each group, N= 56–103) (entire inmunocytochemistry for Mfn1 and DRP1 with control are provided on **Supplementary Figure 3**).

After AβOs characterization, we used PC-12 cells to compare the effects of acute AβOs treatments (1–2 h) on two of the most important proteins related with mitochondrial dynamics: DRP1 and Mfn1 with chronic AβOs treatments (24 h). Using western blot, we evaluated total protein levels of Mfn1 (**Figure 2A**) and DRP1 (**Figure 2B**) in PC12 cells after 1 and 2 h treatments with AβOs (0.5 µM). Western blot analyses showed that Mfn1 protein levels were significantly reduced (**Figure 2C**) after 2 h of acute AβOs treatments compared to control cells (42 ± 11%); while DRP1 (**Figure 2D**) were significantly increased after acute treatments, reaching two-fold increased (1 h: 205 ± 2%, 2 h: 198 ± 3%, **Figure 1D**) but were not seen after prolonged times (24 h) of AβOs treatments (**Supplementary Figure 1**). These results were compared with the effects of FCCP (10 μM, 2 h), which caused a significant increase in the levels of DRP1 (193 ± 27%, **Figure 2D**). Initially, these results suggest that, during mitochondrial dynamics, the fission process could be potentiated by acute exposure to AβOs, and this effect was maintained under chronic conditions. To corroborate these results, we performed immunocytochemistry experiments to evaluate the immunoreactivity of Mfn1 (**Figure 3A**) and DRP1 (**Figure 3B**). Using epifluorescence microscopy, we quantified the total immunoreactivity levels for Mfn1 (48 ± 7%, **Figure 3C**) and found that the fluorescence intensity was significantly reduced at 2 h in line with western blot results. DRP1 immunoreactivity (**Figure 3D**), on the other hand, showed a significantly increase in the fluorescence intensity at 2 h (139 ± 8%, **Figure 3D**), also in line with the observations on western blot. Therefore, these results suggest that acute exposure to AβOs (0.5 μM), induced a fast activation of the fission process as an early event to restore mitochondrial homeostasis, and thus separate the defective mitochondrial population from the functional ones for the maintenance of their vital functions. These changes in the balance of mitochondrial fission and fusion proteins were not seen after prolonged times (6 or 24 h) of AβOs treatments (**Supplementary Figure 1**), suggesting that AβOs could promote fast changes in the mitochondrial network causing and early impact mitochondrial dynamic processes leading to a no-return point in the pathway to cell death.

### Changes in Mitochondrial Network Morphology After AβOs Treatments

**Figure 4:**
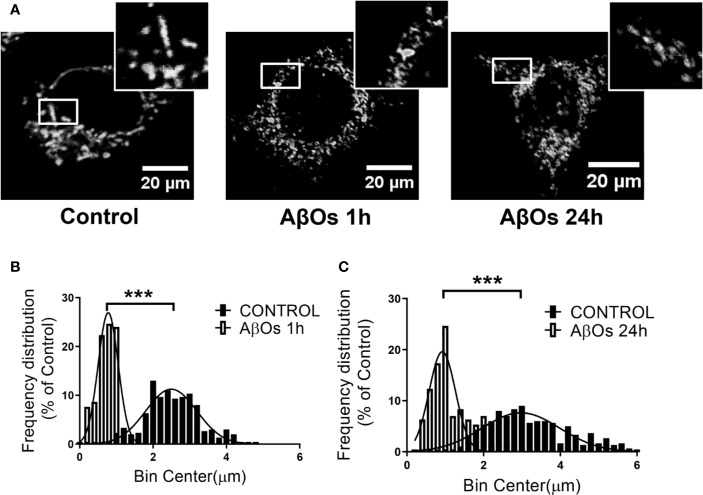
Changes in mitochondrial network size after acute and chronic exposure to AβOs in PC12-cells. **(A)** Confocal images of PC-12 cells showing immunoreactivity of the specific primaryantibody for TOM 20 (white) in control conditions (left panel) and after AβOs treatments (0.5 µM) during 1 and 24 h of incubation (middle panel and right panel, respectively). White squares show a magnification of the soma region of the cell). **(B)** Histogram shows the mitochondrial size distribution after acute and **(C)** chronic treatments with AβOs. Data are represented as mean ± SEM. ***p < 0.001 compared between the control group. Student's t-test followed by Mann-Whitney test was used for all statistical analyses. TOM20, translocase of outer membrane. (n=3, N=18–23) (complete inmunocytochemistry for TOM20 with control are provided on **Supplementary Figure 4**).

**Figure 5:**
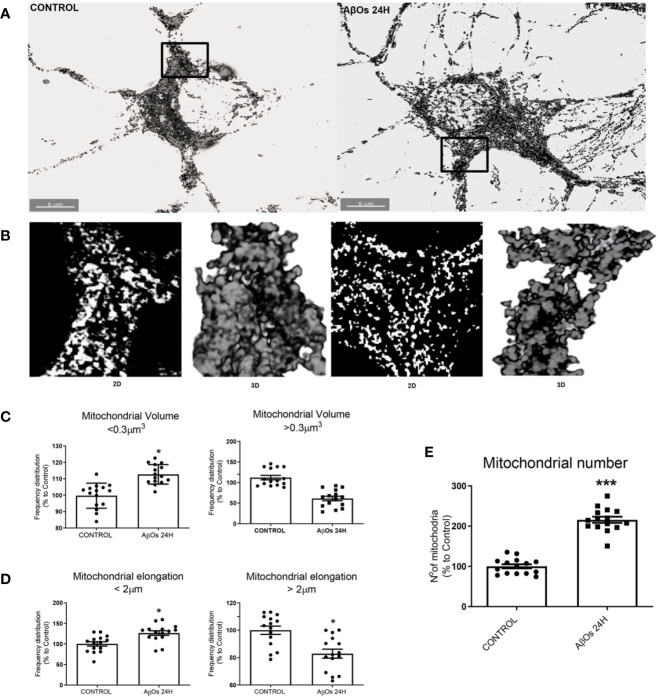
Changes in mitochondrial network size after acute and chronic exposure to AβOs in hippocampal neurons. **(A)** Super-resolution images of hippocampal neurons showing the immunoreactivity for the specific primary antibody for TOM 20 (white) in control condition (left panel) and after 24 h of incubation with AβOs (0.5 µM; right panel). **(B)** Representative 2D and 3D images of mitochondrial population in figure A. **(C)** The data were analyzed as the percentages of number for volume (> and < 0.3 μm3, **C**), length (> and < 2 μm, **D**), and number **(E)** of mitochondrial population in AβOs treated cells compared with untreated cells. Scale bars: 5 μm. Data are represented as mean ± SEM. *p < 0.05, ***p < 0.001 compared between the control group. Student's t-test followed by unpaired t-test was used for all statistical analyses. TOM20, translocase of outer membrane. (n=3, N=15) (complete inmunocytochemistry for TOM20 with controls are provided on **Supplementary Figure 5**).

To confirm our previous observations, we studied the morphology of the mitochondrial network, based on our previous work, where we observed that ATP leakage induced by AβOs causes a decrease on neuronal energetic availability (Saez-Orellana et al., 2016). We evaluated mitochondrial morphometric parameters using a TOM20 marker with confocal and superresolution microscopy to examine if changes in the size and distribution of mitochondria are a consequence of changes in the dynamic's proteins. First, we evaluated morphometric changes in mitochondrial size induced by acute (1 h) and chronic (24 h) AβOs treatments on PC-12 cells using confocal microscopy (**Figure 4A**). We found a significantly reduction in the size of mitochondrial bodies compared to control conditions (C: 2.4 ± 0.04, 1 h: 0.76 ± 0.02, **Figure 4B**), and this fragmented phenotype was also seen significant after chronic treatment with AβOs (24 h: 1.2 ± 0.03, **Figure 4C**) where the mitochondria remained very small and the populations exhibited a granular appearance, instead to the more fibrillary phenotype in control conditions. Next, we evaluated mitochondrial morphometric parameters using a superresolution microscopy in hippocampal neurons (**Figure 5A**), followed by a digital image analysis capable of measuring the area and length of each mitochondria (see digital imaging processing for details) under chronic AβOs treatment. Representative images of zoom regions from 2D and 3D reconstructions of the mitochondrial network by superresolution technique in control (left) and treated (right) conditions are showed on **Figure 5B**, respectively. We evaluated three parameters from these reconstructions: volume (**Figure 5C**), length (**Figure 5D**), and mitochondrial number (**Figure 5E**). We observed that the mitochondria number in AβOs treatment conditions were significantly increased at 24 h (219 ± 9%, **Figure 5E**), which was associated with a significant increase of mitochondrial volume in the smaller mitochondria population (24 h: 112 ± 3%; < 0.3 μm^3^).

Additionally, mitochondria with shorter size were significantly increased with chronic Aβ treatment (24 h), compared to control conditions (24 h:127 ± 5%; < 2 μm, **Figure 5D**, left graph), while largest mitochondria were significantly decreased with chronic Aβ treatment (24 h), compared to control conditions (24 h:83 ± 3%; > 2 μm, **Figure 5D**, right graph). We observed a similar mitochondrial phenotype under acute AβOs treatments (data not shown). Taken together, our hypothesis is that earlier predominance of granular mitochondrial phenotype is associated with an early imbalance in mitochondrial dynamics proteins which appears to be the crucial point to initiate deficits in neuronal metabolism that finally produces synaptic failure and cell death. Curiously, loss of the normal mitochondrial phenotype network after chronic exposure to AβOs did not cause additional changes in the levels of mitochondrial dynamics proteins (data no shown), suggesting that acute loss of these proteins leads to an irreversible mitochondrial fragmented phenotype and could represent a point of no return for the recovery of its function full stop.

### Acute and Chronic AβOs Treatments Induced Changes in Mitochondrial Biogenesis Proteins

**Figure 6:**
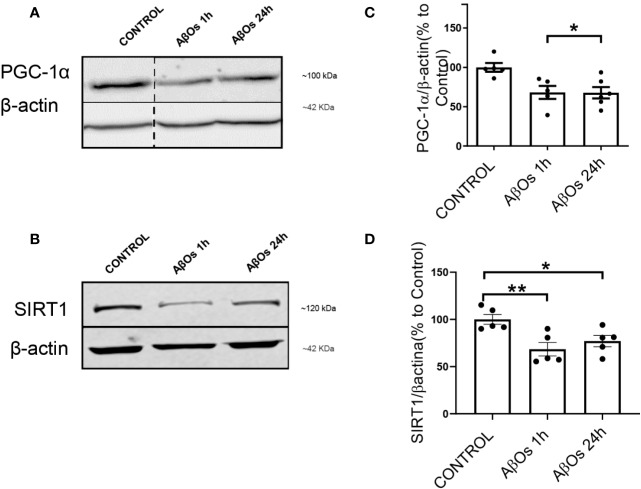
Changes in SIRT1 and PGC-1α levels after exposure to AβOs. **(A)** PGC-1α western blot of PC12 lysates control and treated with AβOs (0.5 μM) for 1 and 24 h. **(B)** SIRT1 western blot of PC12 lysates in the same conditions as A. **(C)** Quantification of PGC-1α protein levels normalized to β actin levels, and **(D)** SIRT1 protein levels under similar conditions as C. Data are represented as mean ± SEM. *p < 0.0, **p < 0.01, compared between the control group. One-way ANOVA with the Dunn's multiple comparisons test was used for all statistical analyses. PGC-1, Peroxisome proliferator-activated receptor gamma coactivator 1-alpha; SIRT1, silent information regulator 2 homolog 1. (n=5–6/group). A dotted line on the western blot indicates different regions of the same gel (original gel blot are provided on **Supplementary Figure 6**).

**Figure 7:**
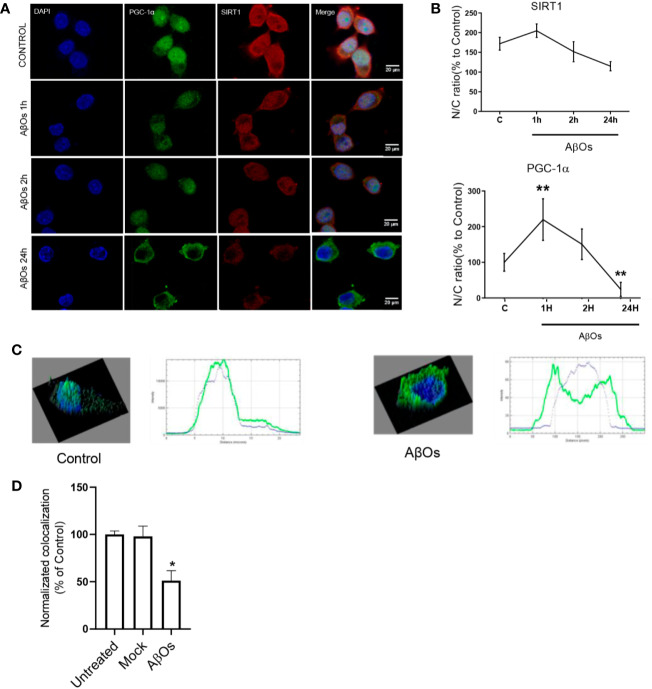
Immunocytochemistry of SIRT1 and PGC-1α in PC12 cells treated with AβOs. **(A)** Representative confocal images of PC 12 cells stained with specific primary antibodies for PGC-1α (Green),SIRT1 (red), and DAPI (blue) in control condition (upper panel), and after AβOs treatments (0.5 µM) during different incubation times (1 and 24 h, lower panels). **(B)** Quantification of the nucleus/cytosol ratio (N/C) for SIRT1 (upper graph) and PGC-1α (lower graph) from images shown in A. **(C)** Surface plot of a representative cell shown in A, correlating spatial distribution (x,y) with fluorescence intensity (z) for DAPI and PGC-1α in control condition (left panel) and after 24 h of treatment with AβOs (right panel). Scale bars: 20 μm. **(D)** Manders colocalization coefficient (MCC) values quantify the different levels of colocalization between PGC-1α and SIRT1. Data are represented as mean ± SEM. *p < 0.05, **p < 0.01, compared between the control group. One-way ANOVA with the Dunn's multiple comparisons test was used for all statistical analyses. PGC-1, Peroxisome proliferator-activated receptor gamma coactivator 1-alpha; SIRT1, silent information regulator 2 homolog 1. (n=3, N=37).

As rescue mechanism, the neurons to protect themselves against AβOs toxicity, are able to activate a biogenesis mechanism to restore the mitochondrial population which is necessary to support key function as synaptic activity (in the case of neurons) or metabolic activity (in the case of common cells). Thus, there is a possibility that the irreversible effects of AβOs could be associated with an impediment of the mitochondria to repair and/or restore their intracellular network. Therefore, to evaluate this aspect we examined the status of the key mitochondrial biogenesis regulators: PGC-1α (**Figure 6A**) and SIRT1 (**Figure 6B**). Changes in the total levels of these proteins were evaluated to determine if the cells could activate the generation of new functional mitochondria after AβOs treatment in response to the toxic action of Aβ and avoid the generation of the fragmented phenotype seen previously. However, after acute and chronic AβOs exposure in PC12 cells (1–24 h), western blot studies showed a significantly decreased in PGC-1α levels (1 h: 68 ± 8%; 24 h: 67 ± 7%, **Figure 6C**). Similarly, SIRT1 were decreased (**Figure 6B**) after acute and chronic treatments with AβOs compared to control cells (1 h: 68 ± 7%; 24 h: 77 ± 6%, **Figure 6D**). This data is in line with immunocytochemistry and confocal microscopy results that informed changes in the intracellular distribution and immunoreactivity of PGC-1α and SIRT1 in PC12 cells (**Figure 7A**). Interestingly, after 24 h of AβOs exposure, nucleus/cytosol ratio(N/C) of PGC-1α were significantly decreased compared to control conditions (24 h ± 4%, **Figure 7B**, lower graph), with a change in their spatial distribution of immunoreactivity (x,y) (**Figure 7C**, surface plots and plot profiles), suggesting that in cells exposed to AβOs the mechanisms for repair and maintenance of mitochondrial population and functions are defective. However, we did not find any statistically significant changes in SIRT1 total levels or distribution after acute or chronic treatment with AβOs (**Figure 7B**, upper graph).

**Figure 8:**
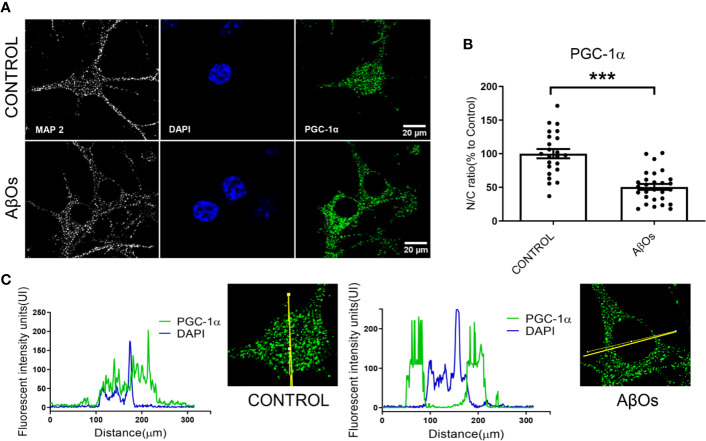
Immunocytochemistry to evaluate the PGC-1α distribution in hippocampal neurons treated with AβOs. **(A)** Representative confocal images of immunoreactivity for specific primary antibodies for PGC-1α (Green) and DAPI (blue) in PC-12 cells in control condition (upper panel) and after 24 h of AβOs treatment (0.5 µM, lower panel). **(B)** Quantification of the nucleus/cytosol (N/C) ratio for images shown in A. **(C)** Plot profiles for images showing the intensity of fluorescence in control condition (left panel) and after 24 h treatment with AβOs (right panel) of images obtained in A. Scale bars: 20 μm. Data are represented as mean ± SEM. ***p < 0.001 compared between the control group. One-way ANOVA with the Dunnett's multiple comparisons test was used for all statistical analyses. PGC-1α, Peroxisome proliferator-activated receptor gamma coactivator 1-alpha; SIRT1, silent information regulator 2 homolog 1. (n=3, N=23–28) (hinmunocytochemistry with controls ares provided in **Supplementary Figure 8**).

We replicated the same experimental conditions in hippocampal neurons to evaluate the effects of AβOs exposure at 24 h (**Figure 8A**). Using confocal microscopy images, observed a significantly decreased on the nucleus/cytosolic ratio of PGC-1α in AβOs-treated neurons (24 h: 51 ± 8%, **Figure 8B**). The analysis of the distribution of immunofluorescence with plot profile tools, shown that the fluorescence profile for PGC-1α that overlaps with the fluorescence profile for DAPI nuclear stain in control conditions (**Figure 8C** left graph); while, in the treatment with AβOs the fluorescence profile for PGC-1α shown increased fluorescence intensities in non-DAPI areas (**Figure 8C**, right graph) suggesting that after AβOs treatment this protein is no longer available in the nucleus to support mitochondrial biogenesis. To complement these results, we wanted to evaluate the effects of AβOs on a more physiological and conserved matrix.

**Figure 9:**
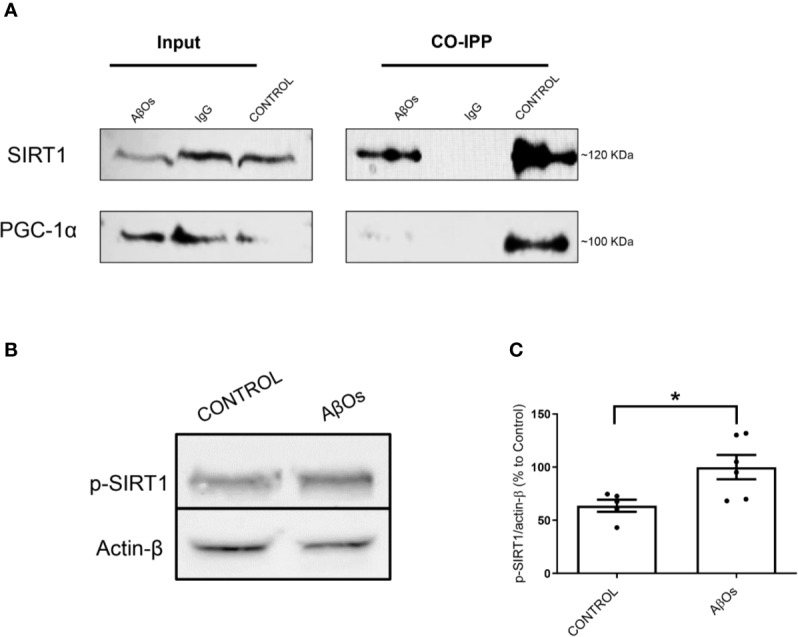
Changes in interaction between the PGC-1α/SIRT1 and association with the levels of p- SIRT1 after chronic treatment with AβOs. **(A)** Representative western blot for PGC-1α (91 kDa) and SIRT1 (120 kDa) in PC-12 cells in control condition (Iane 1) and after chronic treatment with AβOs (0.5 μM; Iane 3). Lane 2 is the IgG control. Co-immunoprecipitation of SIRT1 with PGC-1α are shown in lanes 4, where we can see untreated cells, lane 5 corresponds a IgG control, and lane 6 corresponds to AβOs treatment **(B)** western blot of whole cell lysates of PC12 cells treated with AβOs (0.5 μM) for 24 h showing p-SIRT1 levels. **(C)** Quantification of p-SIRT1 levels normalized to β actin. Data are represented as mean ± SEM. *p < 0.05, compared between the control group. Student's t-test followed by unpaired t-test was used for all statistical analyses. PGC-1α, Peroxisome proliferator- activated receptor gamma coactivator 1-alpha; SIRT1, silent information regulator 2 homolog 1 (n=3/group) (Original blots are provided in **Supplementary Figure 9**).

These results suggest that mitochondrial dysfunction and consequent neuronal failure and synaptic silencing could be associated mainly to changes in PGC-1α more than SIRT-1. Even though our western blot data showed a significantly decrease in SIRT1 at 1 and 24 h, we did not observe any changes regarding SIRT1 nucleus/cytosol ratio in PC-12 cells (**Figure 7**; **Supplementary Figure 7**). However, as mentioned previously, the interaction between PGC-1α and SIRT-1 is fundamental to the biogenesis process. Thus, we studied if the accumulation of PGC-1α in the cytosol was related to changes in the interaction with SIRT1 that could alter the activation of mitochondrial biogenesis. Using co-immunoprecipitation, we observed that the interaction between both proteins was lost after 24 h of AβOs treatment (**Figure 9A**), confirming that PGC-1α is unable to access to the nucleus and activate the mitochondrial biogenesis process, which agrees with our previous results. Additionally, we evaluated the mechanism by which AβOs reduced the level of SIRT1 proteins. To do this, we exposed PC-12 cells and hippocampal neurons to AβOs and evaluated the phosphorylation status of SIRT1 at its serine 47 residue, since this phosphorylation induces ubiquitination and proteasome-dependent degradation in SIRT1 (Gao et al., 2011). Western blot analysis (**Figure 9B**) showed that the levels of pSIRT1 were significantly increased in PC-12 cells treated with AβOs compared to control (157 ± 18%, **Figure 9C**).

**Figure 10:**
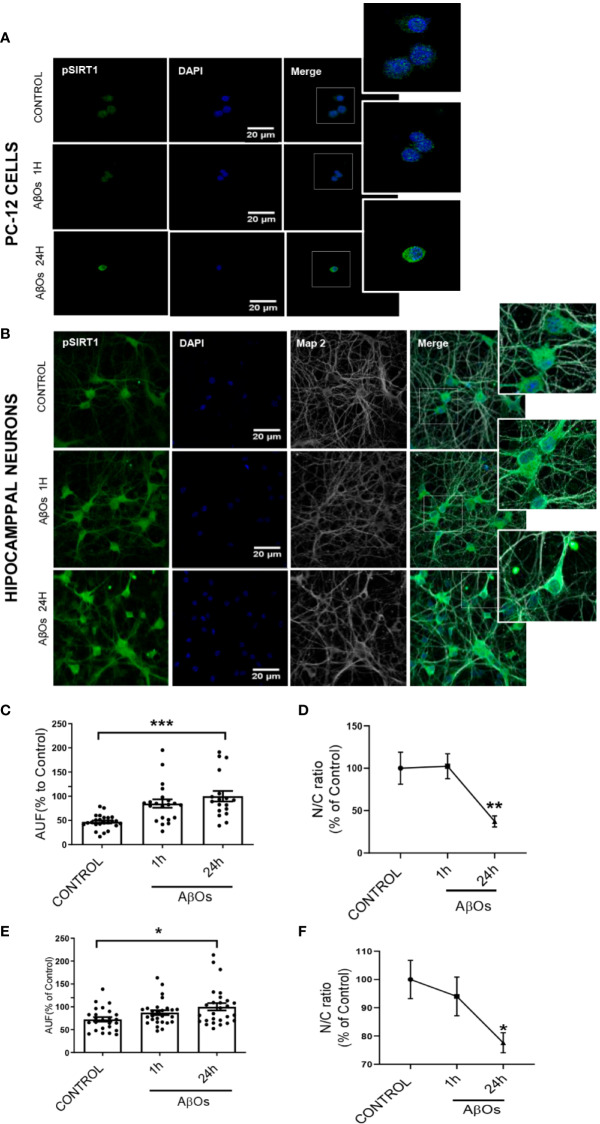
Immunocytochemistry to evaluate p-SIRT1 distribution in PC12 cells and hippocampal neurons treated with AβOs. Representative confocal images of **(A)** PC12 cells and **(B)** hippocampal neurons showing the immunoreactivity of specific primary antibodies for ph-SIRT1 (green) and DAPI (blue) in control condition (upper panel) and after AβOs treatment (0.5 µM) during different incubation times (1, 24 h lower panels). **(C)** Quantification of the total levels of ph-SIRT1 (lower left panel) and Quantification of the relation of nuclear and cytosolic (N/C) of p-SIRT1 in PC-12 cells **(D)**. **(E)** Quantification of the total levels of ph-SIRT1 (left corner panel) and quantification of the relation of nuclear and cytosolic (N/C) of p-SIRT1 in hippocampal neurons **(F)** expressed as percentage of control. Scale bars: 20 μm. Data are represented as mean ± SEM. *p< 0.05, **p < 0.01 ***p < 0.001 compared between the control group. One-way ANOVA with the Dunnett's multiple comparisons test was used for all statistical analyses. SIRT1, silent information regulator 2 homolog 1. [n=3, N=18–27 (Complete inmunocytochemistry with controls are provided in **Supplementary Figure 10**)].

Using immunocytochemistry, we studied the changes on p-SIRT1 in PC12 Cells (**Figure 10A**) and hippocampal neurons (**Figure 10B**). We found that despite the increase on p-SIRT1 fluorescence intensity (**Figure 10C**), the nucleus/cytoplasm ratio of p-SIRT1 decreased in a time-dependent manner with respect to control in PC12 cells (**Figure 10D**) (24 h: 47 ± 5%). In parallel, hippocampal neurons (**Figure 10B**), also shown a moderate increase fluorescence intensity (**Figure 10E**), and a significantly decrease of nucleus/cytoplasm ratio of p-SIRT1 under the same AβOs treatment (24 h: 73 ± 8%, **Figure 10F**). Taken together, our data suggest that p-47 phosphorylation induce a SIRT1 structural change that may precludes its translocation to the nucleus to carry out its function.

**Figure 11:**
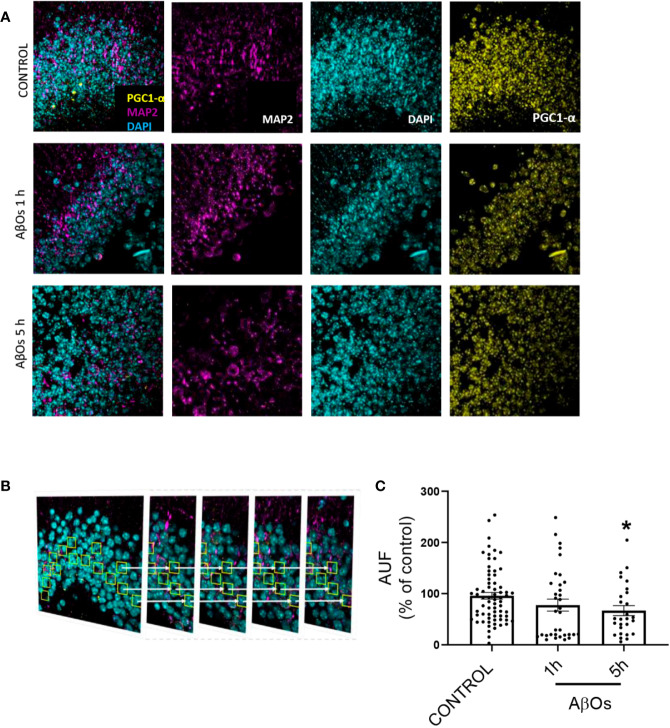
Immunohistochemistry of PGC-1α after acute and sub-chronic treatments of AβOs in mice hippocampal slices. **(A)** Maximum intensity projections of confocal images showing neurons from the granular layer of the dentate gyrus in mice hippocampus stained for: PGC-1α (yellow), MAP2 (violet, for neurons), and DAPI (cyan, for nucleus). **(B)** Maximum intensity projections of stacked confocal images showed in **(A)**. **(C)** Normalized quantification of the fluorescence intensity for PGC-1α normalized with respect to MAP2 immunofluorescence in control condition and after AβOs (0.5 μM) exposure for 1 or 5 h. Scale bars: 5 μm. Data analyzed using one way ANOVA with Kruskall-Wallis test and Dunn's Multiple Comparison test. (*p < 0.05 vs control nroi: 462/nslices: 75, AβOs 1H nroi: 664/nslices: 37, AβOs 5H nroi: 977/nslices: 30/per each independent experiment, n=3).

Finally, we obtained hippocampal slices from 9 months old mice, exposed them to AβOs for 1–5 h and then performed immunohistochemistry (**Figure 11A**). Using confocal microscopy, we observed similar phenomena than in cultured cells (see **Figure 6**), i.e. a significant decrease in PGC-1α immunoreactivity after AβOs treatments. To further examine this result, we analyzed a stack of images for each condition to evaluate the immunoreactivity of PGC-1α in several planes of the same tissue (**Figure 11B**). The results showed that PGC1α immunoreactivity was reduced by AβOs treatments at 5 h 407 (**Figure 11C**, **Supplementary Figure 11**, and **Supplementary Figure 12**).

## Discussion

The neuronal mitochondrial network is regulated by a dynamic process to support high metabolic activity of the brain cells, especially the ATP demand and Ca^2+^ handling (Alonso et al., 1999) that support relevant processes such as synaptic communication, neurotransmitter synthesis and others (Kann and Kovács, 2007). Mitochondrial function depends on the correct performance of mitochondrial dynamics and mitochondrial biogenesis, which coordinates the formation of new functional mitochondria, and is an important defense mechanism for cells to survive from mitochondrial damage (Hackenbrock et al., 1971; LC et al., 2011), then the loss of functional mitochondria could be a very sensitive problem to the neurons. It has been observed that mitochondrial dysfunction is one of the earliest and most prominent features of AD (Schmitt et al., 2011). Mitochondrial cascade hypothesis in AD proposes that a mitochondrial perturbation precede the synaptic damage, the neuronal cell death, and the deficits in learning/memory ability in AD (Swerdlow et al., 2010; Swerdlow, 2018). A new approach for the treatment of AD based on the preservation of the mitochondrial structure as a target (Course and Wang, 2016; Arrazola et al., 2017) opens a new line of study, and according with our data PGC1-α could be a interesting candidate.

Several studies postulate that mitochondrial failure may occur from a dysfunction in the mechanisms involved in mitochondrial turnover (the balance between organelle biogenesis and dynamics), which may also help delineate the underlying causes in age-related dysregulation of mitochondria in damaged neurons that are not available to support the metabolic demand of the synapse (Bonda et al., 2010). In the AD case, the lost on neuronal connectivity and synaptic failure mediated mainly by AβOs toxicity (Ferreira and Klein, 2011; Saez-Orellana et al., 2016), has been correlated with an early mitochondrial function loss (Eckert et al., 2010; Mendoza et al., 2014), cell damage (Reddy et al., 2012), and cognitive failure (Kimata et al., 2012). However, the mechanisms by which AβOs signaling promotes neuronal cell death and mitochondrial dysfunction are unclear (Swerdlow, 2018). The property of PGC-1α to interact with multiple DNA-binding transcription factors (TFs) to regulate the mitochondrial biogenesis and dynamic it allows maintenance of mitochondrial protein levels and other relevant proteins such as calmodulin-dependent kinase (CaMK), calcineurin (CN), PPARs, AMP-activated kinase (AMPK), SIRT1, ERR regulator in muscle 1, and retinoid X receptor (RXR) (Dorn et al., 2015), reinforce our idea about the consideration as new molecular target for modulation. During PPAR/PGC-1α interaction, the stimulation can rescue mitochondrial dysfunction and induces improvement in mitochondrial dynamic events (Fuenzalida et al., 2007). In this context expression of several nuclear and mitochondrial genes, Drp1, Mfn1 and mitochondrial transcription factor A (TFAM), is necessary for mitochondrial biogenesis (Westermann, 2010). Indeed, agonists for both PPARγ and α can prevent Aβ-associated mitochondrial dysfunction through a PGC-1α-dependent mechanism (Zolezzi et al., 2017), and it's able to promote axonal growth, reduce oxidative stress, and improve brain clearance of toxic Aβ peptide (Puigserver et al., 1998; Zolezzi et al., 2017).

Taken together, any alterations on its role as fine tuner of mitochondrial biogenesis could be crucial for neuronal functions. Therefore the possibility to understand the early changes in this factor that can induce the loss of balance between fusion and fission may be considered as early market of neuronal damage by toxic agents, in this case amyloid β peptide. Several studies have reported that the level of PGC-1α evidently decreases in association with diminished mitochondrial density in the brains of AD patients (Qin et al., 2009). Indeed PGC-1α able to preserve synaptic function in animal models of AD (Halagappa et al., 2007), acting as a possible target for therapeutic intervention in AD (Sweeney and Song, 2016).

Upon the occurrence of cellular metabolic stress conditions, NAD^+^/NADH ratio increases, promoting SIRT1 activation and transcriptional activity of PGC-1α by deacetylating at least one of its 13 acetylated lysine residues (Nogueiras et al., 2012). SIRT1 deacetylase activity has been linked to a neuroprotective effect in murine models of AD (Kim et al., 2007). Specifically, activation of SIRT1 manages to improve dendritic branching and neuronal function by inactivating the RhoA/ROCK pathway (Codocedo et al., 2012; Godoy et al., 2014). Indeed, positive modulation of the expression levels and activity of SIRT1 successfully improved energy expenditure in several animal models for neurodegenerative diseases associated with mitochondrial dysfunction (Donmez, 2012), and in case of AD it is able to diminish the memory deficits (Kim et al., 2007), but the mechanism that confers neuroprotection is still poorly understood.

In our work, we provide evidence about a biphasic behavior of PGC-1α/SIRT1 in a time-depend Aβ toxicity. Our first experimental evidence, as consequences of acute AβOs exposure, was the imbalance in the fission/fusion proteins (**Figures 2** and **3**) that induced a shift in the equilibrium to granular or fractioned mitochondrial phenotype (**Figures 4** and **5**), suggesting that the energetic pathway into the neurons it was interrupted. We expected that mitochondrial phenotype promotes an increase in the activation of signaling molecules for mitochondrial biogenesis as compensatory biological response to produce more functional and healthy mitochondria (a quantitative increase to overcome a qualitative deficiency, due to alterations in mitochondrial proteins that are either nuclear or mitochondrial encoded). However, we found that PGC-1α changed its subcellular distribution from predominant nuclear distribution in control conditions, to cytosolic distribution of PGC-1α (**Figures 7C** and **8C**), after chronic treatment with AβOs. The options to explain that PGC-1α was unable to translocated to the nucleus could be linked with changes on the levels of the protein, but without apparent differences on its immunoreactivity, the possibility that the changes from the acetylated form to deacetylated form, suggest that the option of an alteration with other elements in the signaling pathway like SIRT1. When attempting to elucidate this phenomenon, we evaluated the phosphorylated form of p-SIRT (Ser 46, 47 in humans) that regulates the SIRT1 availability. Phosphorylation of SIRT1 by JNK1 as a result of cell stress induced a brief activation of the SIRT1 function, followed by the degradation of SIRT1 by the proteasome due to its ubiquitination (Gao et al., 2011).

Under chronic treatments of AβOs, total levels of pSIRT1 were increased (**Figure 9C**), and there was a significant decrease in its nucleus/cytosol ratio (**Figure 10**) presenting a behavior like cytosolic PGC-1α (**Figure 7B**), was not observed for total SIRT1 distribution (**Figure 7B**). We have found that an increased in p-SIRT1(Ser 46), could be related with a novel mechanism induced by chronic treatments of AβOs, which also altered SIRT1 interaction with PGC-1α. We propose that this pathway could be directly associated to overactivation upon JNK1, and SIRT1 ubiquitination and degradation according with the evidence provided by Okazawa and Gadhave groups (Okazawa and Estus, 2002; Gadhave et al., 2016).

**Figure 12:**
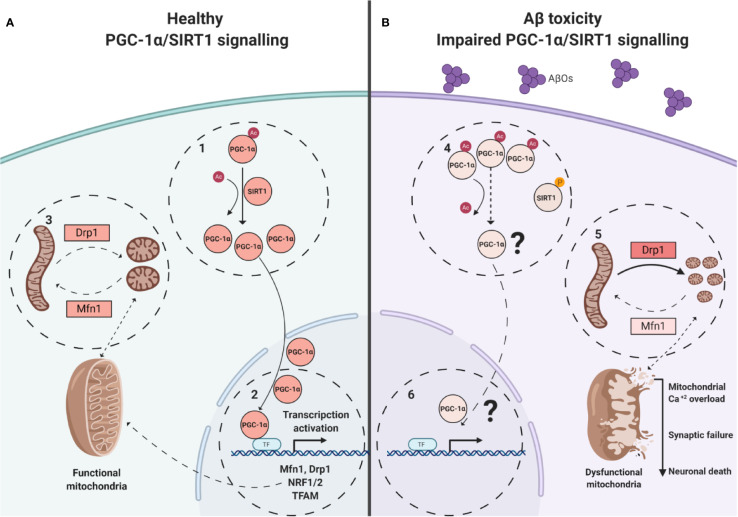
Model of alterations induced on SIRT1/PGC-1α pathway by AβOs. **(A)**. Healthy neuronal conditions were energy depletion and/or decreased catabolic rates can be sensed by SIRT1 promoting the PGC-1α deacetylation (1). Transcription and co-activates of factors like NRF- 1/2 for the expression of nuclear-encoded mitochondrial genes and dynamic mitochondrial proteins, requires of PGC-1α translocation (2). Coordinated mitochondrial dynamics (3, fission/fusion), depends of adequate expression of Mfn1, Drp1. **(B)**. In AβOs treated neurons, the PGC-1α is unable to be deacetylated and to translocate to the nucleus (4). The expression of key genes is loss (5), and imbalance between fusion and fission to promote the granular mitochondrial phenotype (6) and neuronal death.

We performed morphologic analyses of the mitochondrial network in PC-12 cells, to corroborate our findings and the data showed that there were early alterations in mitochondria shape after treatment with AβOs (**Figures 4** and **5**). There was a clear granular phenotype that persisted even at chronic exposure times. At the chronic times, the total levels of Drp1 and Mfn1 did not change (**Supplementary Figure 1**). These findings are in line with the study that identify a previously unknown mitochondrial fission arrest phenotype that resulted in elongated interconnected organelles called “mitochondria-on-a-string” (MOAS) (Zhang et al., 2016). However, after chronic AβOs treatment (**Figures 6** and **11**), we found a significantly reduction in the total levels of both PGC-1α and SIRT1, but only PGC-1α presented an alteration in the nucleus/cytosol ratio showing an increase in its cytosolic distribution, reinforcing the idea that PGC-1α it has been “sequestered” in the cytosol, and this could be explained by a significant decrease on the interaction between SIRT1 and PGC-1α after chronic exposure to AβOs (**Figures 9A** and **12**). Several evidence suggest the existence of an orchestration between mitochondrial biogenesis and mitochondrial dynamics. For instance, these findings described above indicate that mitochondrial dynamics can affect mitochondrial biogenesis. PGC-1α-deficient mice showed an altered mitochondrial morphology including fragmentation and elongation and defects in proper mitochondrial dynamics in which the levels of Mfn1 were downregulated (Uittenbogaard and Chiaramello, 2014). In summary, recent data have implicated the existence of cross-regulatory circuits that coordinate both mitochondrial dynamics and mitochondrial biogenesis (Martin et al., 2014; Dorn et al., 2015; Song et al., 2015).

In this study we describe an important link between SIRT1/PGC-1α downregulation and the fast-morphological changes observed on mitochondria networks, promoting by a misbalance on DRP1/Mfn1 in a Aβ toxicity model (**Figure 12**). The “sequestered” PGC-1α in the cytosol could represent the non-return point for the neuron on the amyloid β toxicity. This study suggests a possible new approach for the treatment of AD, based on the preservation of the mitochondrial structure which open a new space of study centered on the modulation of PGC-1α/SIRT1 signaling, by PGC-1α deacetylation promotors, as new pharmacological tools to promote neuroprotection.

## Data Availability Statement

All datasets generated for this study are included in the article/**Supplementary Material**.

## Ethics Statement

All procedures related with animal management and tissue isolation were done following NIH (USA) and CONICYT guidelines and with protocols approved by the Bioethical Committee of the Universidad de Concepcion

## Author Contributions

JP and JF designed the study. JP, PG, OR-M and JG contributed to the sample preparation, data collection and analysis. JP, JF, CM-M, GY, GM-C, PC, LG, JS, and ET contributed to write the manuscript. JP, TS-G, and MC directed the experiments. All authors reviewed the manuscript.

## Funding

This work was supported by Fondecyt 1161078 (JF), 1200908 (JF), and Conicyt 21141247 (JP).

## Conflict of Interest

The authors declare that the research was conducted in the absence of any commercial or financial relationships that could be construed as a potential conflict of interest.

## Abbreviations

AD, Alzheimer's disease; AMPK, AMP-activated kinase; APP, Amyloid precursor protein; Aβ, β-amyloid peptide; AβOs, Solubles Oligomers of Aβ peptide; CaMK, calmodulin-dependent kinase; CN, calcineurin; DRP1, Dynamin-related protein; FCCP, Uncoupler p-trifluoromethoxyphenyl-hydrazone; Fis1, Mitochondrial fission 1 protein; GCN5, N-acetyltransferases; HDAC, Histone Deacetylases; JNK1, C-Jun N-terminal kinases; Mfn1, Mitofusin 1; NRF, Nuclear respiratory factor; OPA1, Mitochondrial dynamin like GTPase; PGC-1α, Peroxisome proliferator-activated receptor gamma coactivator 1-alpha; PPARγ, Peroxisome proliferator-activated receptor gamma; RXR, retinoid X receptor; SIRT1, silent information regulator 2 homolog 1; TFAM, transcription factor A; TOM20, Mitochondrial import receptor subunit TOM20.
