# Supplementary material for: Changes in PGC‐1α/SIRT1 Signaling Impact on Mitochondrial Homeostasis in Amyloid-Beta Peptide Toxicity Model
Source: Front Pharmacol. 2020 May 21;11:709. doi: 10.3389/fphar.2020.00709 (PMC7261959; doi:10.3389/fphar.2020.00709)
Supplement: Supplementary file 1 [file DataSheet_1.pdf]

Supplementary Figures

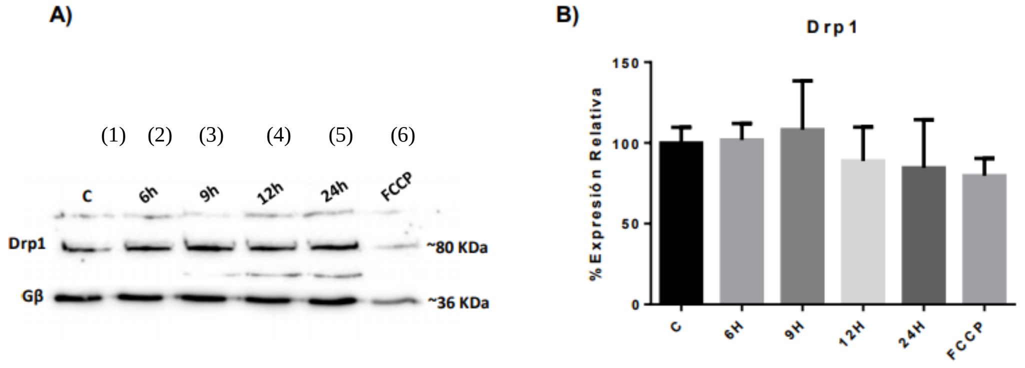

Supplementary Figure 1. Temporal curve of Drp1 expression after treatment with AβOs. (A) Representative image of the Western blot performed on lysates of PC12 cells where the Drp1 protein was immunodetected after treatment with 0.5 μM AβOs for 6 (lane 2), 9 (lane3), 12 (lane 4) and 24 h (lane 5) together with a 10 μM FCCP(lane 6), comparated to untretated cells (lane 1). (B) Graph of the quantification of the relative expression of Drp1, with respect to the Gβ charge control, expressed as a percentage of the control (n = 3, of three independent experiments).

A

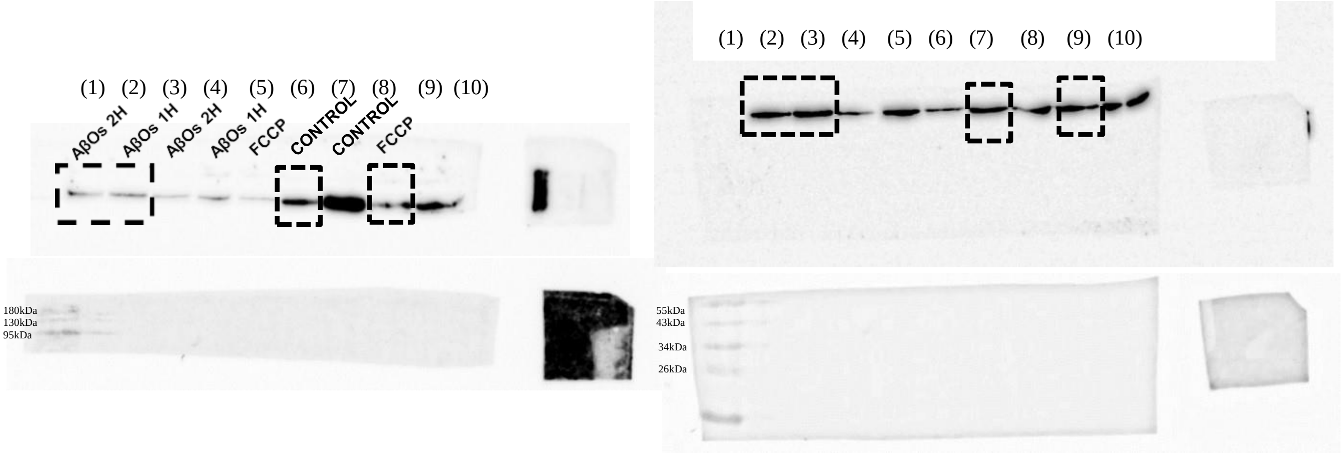

B

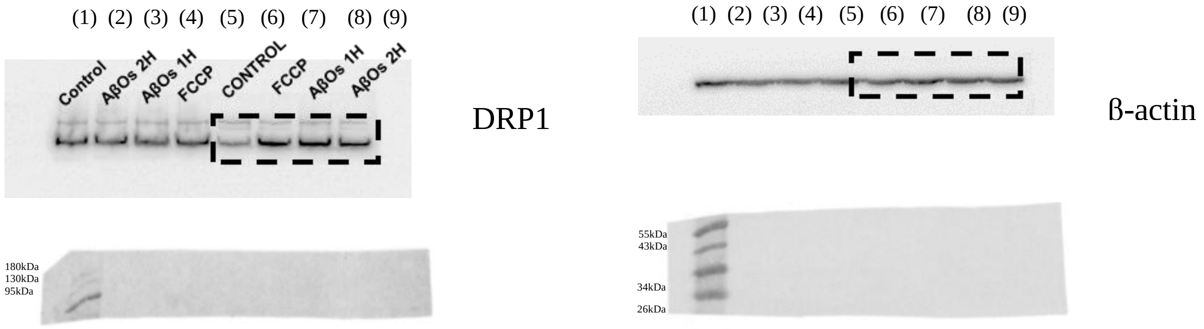

Supplementary Figure 2. A. Original gels for Figure 2A(shown in dotted line) to Mfn1 and its MW channel (left panel), and beta actin and its MW channel (right panel). The lanes correspond to (1) Page ruler (2) AβOs 2h (3) AβOs 1h (4) AβOs 2h (5) AβOs 1h (6) FCCP (7) Control (untreated) (8) Control(mock) (9) FCCP(10) AβOs 24h (9) Page ruler . We use beta actin as a loading control (Figure 2A is shown in dotted lines). B Original gels for Figure 2B (shown in dotted line) to DRP1 and its MW channel (bottom panel), and beta actin and its MW channel (bottom panel). The lanes correspond to (1) Page ruler (2) Control (mock) (3) AβOs 2h (4) AβOs 1h (5) FCCP (6) Control (untreated) (7) FCCP (8) AβOs 1h (8) AβOs 2h. We using beta actin as a loading control (Figure 2A is shown in dotted lines).

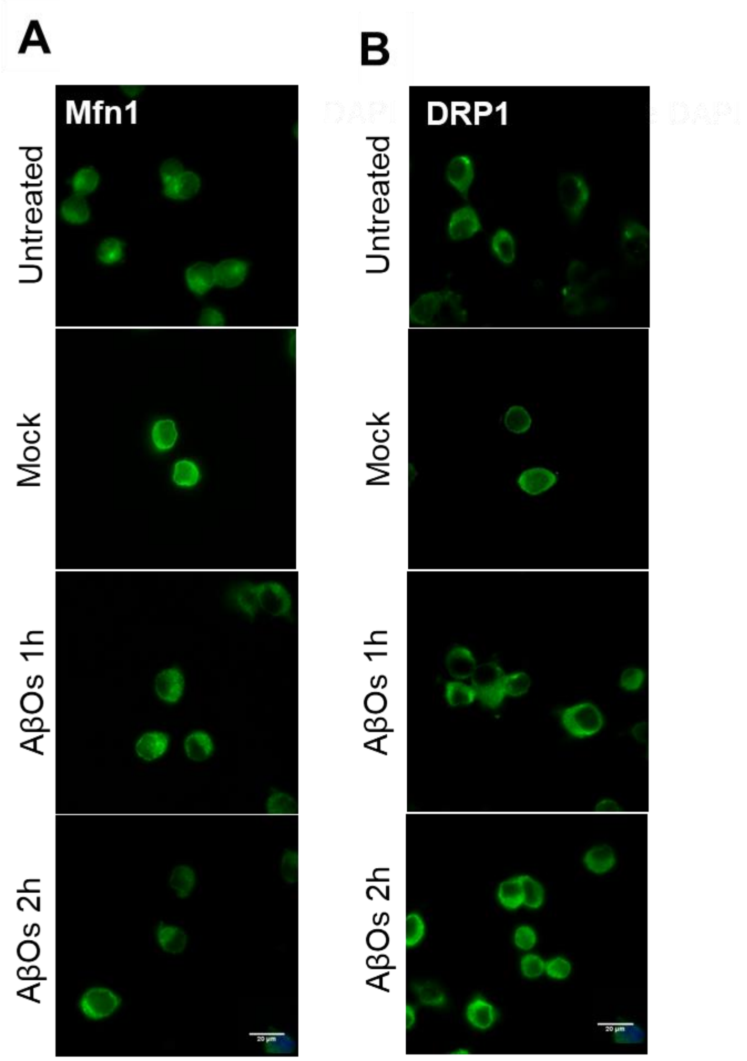

Supplementary Figure 3. Effects of AβOs on immunoreactivity of proteins that regulate mitochondrial dynamics. Representative epifluorescence images of (A) Mfn1 and (B) DRP1 immunoreactivity in PC-12 cells control (untreated and mock of oligomerization) and treated with AβOs (0.5 μM) for 1 and 2 h.

In review

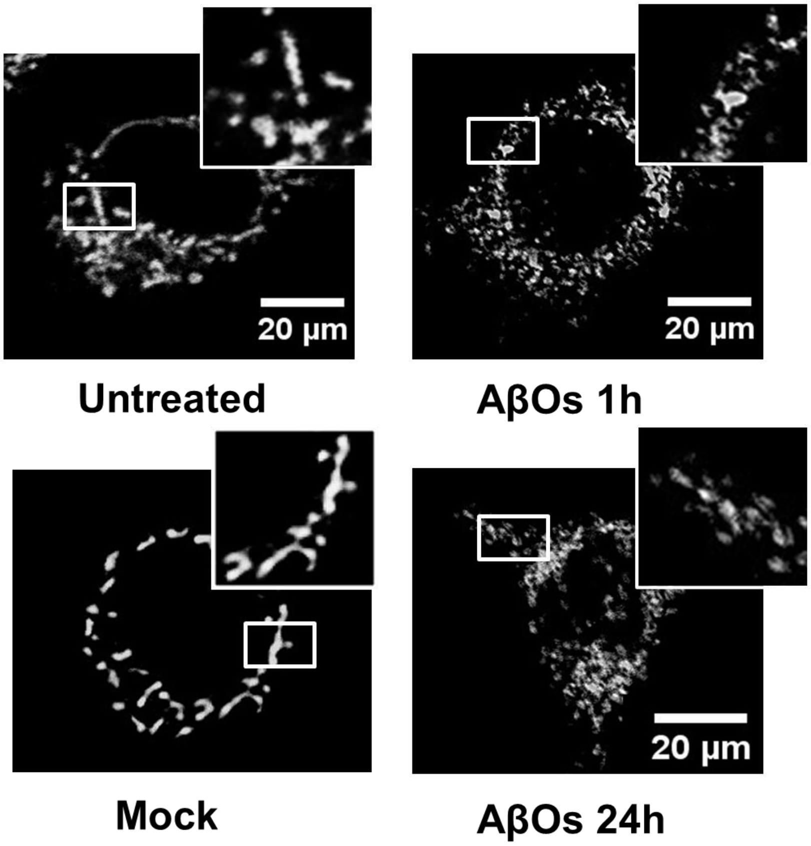

Supplementary Figure 4. Changes in mitochondrial network size after acute and chronic exposure to AβOs in PC12-cells. (A) Confocal images of PC-12 cells showing immunoreactivity of the specific primary antibody for TOM 20 (white) in control conditions (left panel untreated and mock oligomerization) and after AβOs treatments (0.5 µM) during 1 and 24h of incubation (right panel, respectively). White squares show a magnification of the soma region of the cell).

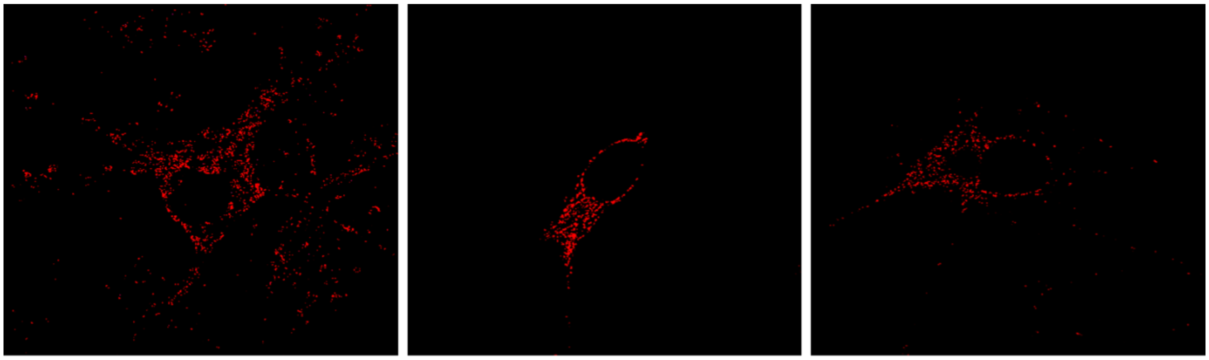

Supplementary Figure 5. Changes in mitochondrial network size after chronic exposure to AβOs in hippocampal neurons. (A) Confocal images of hippocampal neurons showing immunoreactivity of the specific primary antibody for TOM 20 (red) in control conditions (left and middle panel untreated and mock oligomerization) and after AβOs treatments (0.5 μM) during 24h of incubation (right panel, respectively).

A

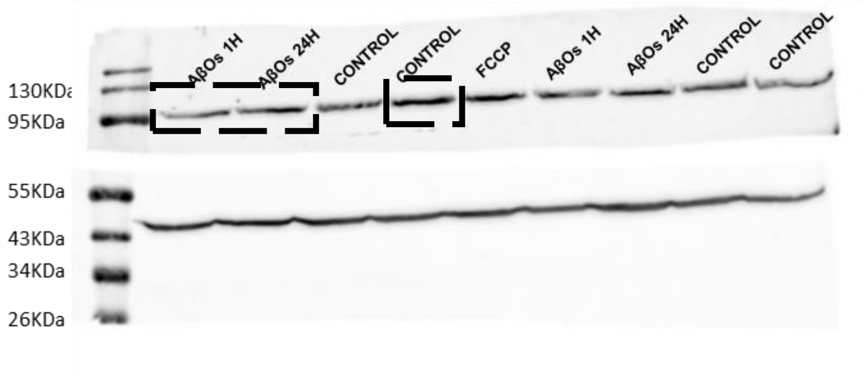

B

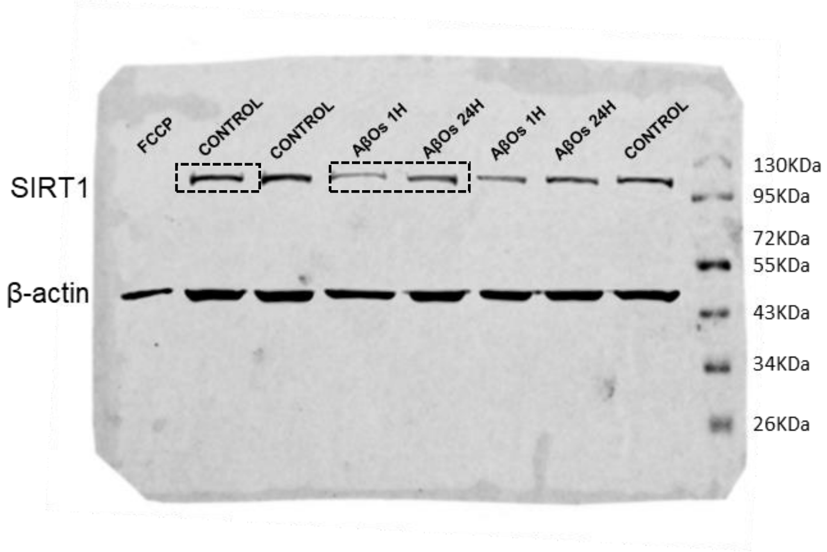

Supplementary Figure 6. A. Original gels for Figure 6A (shown in dotted line) to PGC-1α (left panel), and beta actin (right panel). The lanes correspond to (1) Page ruler (2) AβOs 1h (3) AβOs 24h (4) Control (mock oligomerization) (5) Control (untreated) (6) FCCP (7) duplicate of AβOs 1h (8) duplicate of AβOs 24h (9) Control (10) duplicate of Control. (Figure 6A is shown in dotted lines). B. Original gels for Figure 6B (shown in dotted line) to SIRT1 (left panel) and beta actin (right panel). The lanes correspond to (1) FCCP (2) Control (untreated) (3) Control(mock oligomerization) (4) AβOs 1h (5) AβOs 24h (6) duplicate of AβOs 1h (7) duplicate of AβOs 24h (8) Control (9) Page ruler. (Figure 6B is shown in dotted lines).

A

B

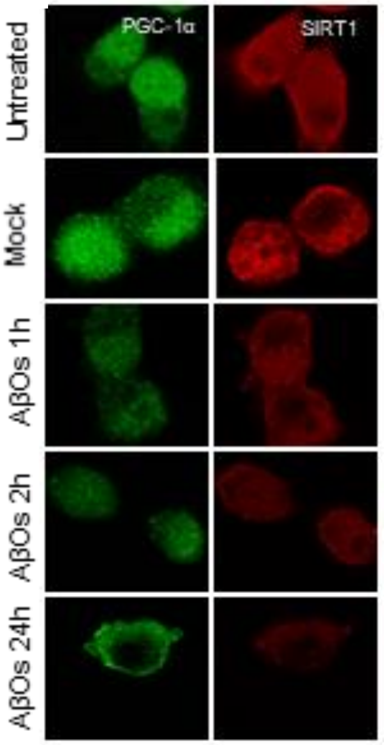

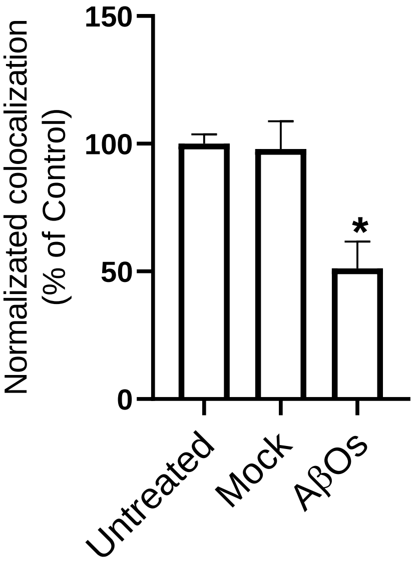

Supplementary Figure 7. Immunocytochemistry of SIRT1 and PGC-1α in PC12 cells treated with AβOs. (A) Representative confocal images of PC 12 cells stained with specific primary antibodies for PGC-1α (Green) and SIRT1 (red) in control condition (untreated, upper panel, mock oligomerization middle panel), and after AβOs treatments (0.5 μM) during different incubation times (1 and 24h, middle and lower panels). B Manders colocalization coefficient (MCC) values quantify the different levels of colocalization between PGC-1α and SIRT1 (refers to Figure 7)

A

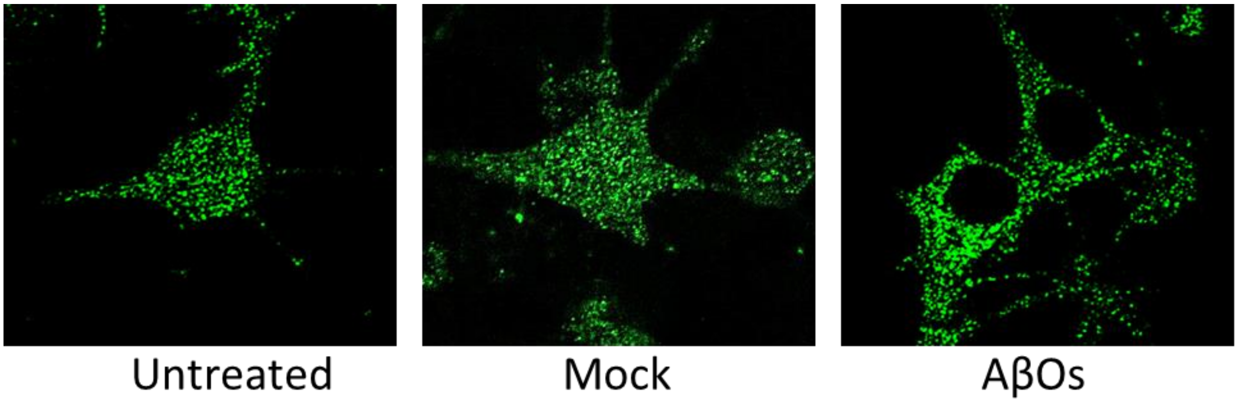

B

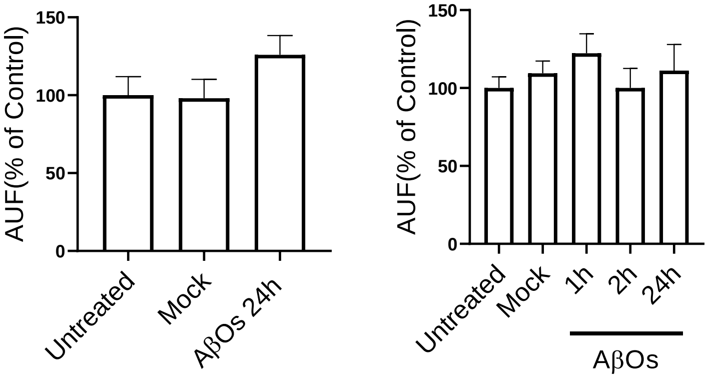

Supplementary Figure 8. A. Immunocytochemistry of SIRT1 and PGC-1α in hippocampal cells treated with AβOs. (A) Representative confocal images of hippocampal neurons stained with specific primary antibodies for PGC-1α (Green) in control condition (untreated left panel, mock oligomerization middle panel), and after AβOs treatments (0.5 µM) during incubation (24h, right panel). **B.** Quantification of the total levels of PGC-1α, left panel for hippocampal neurons (from Fig 8 to PGC-1α) and right panel for PC-12 cells (from Fig 7 to PGC-1α). Mean ± SEM, n=3, N=18-37.

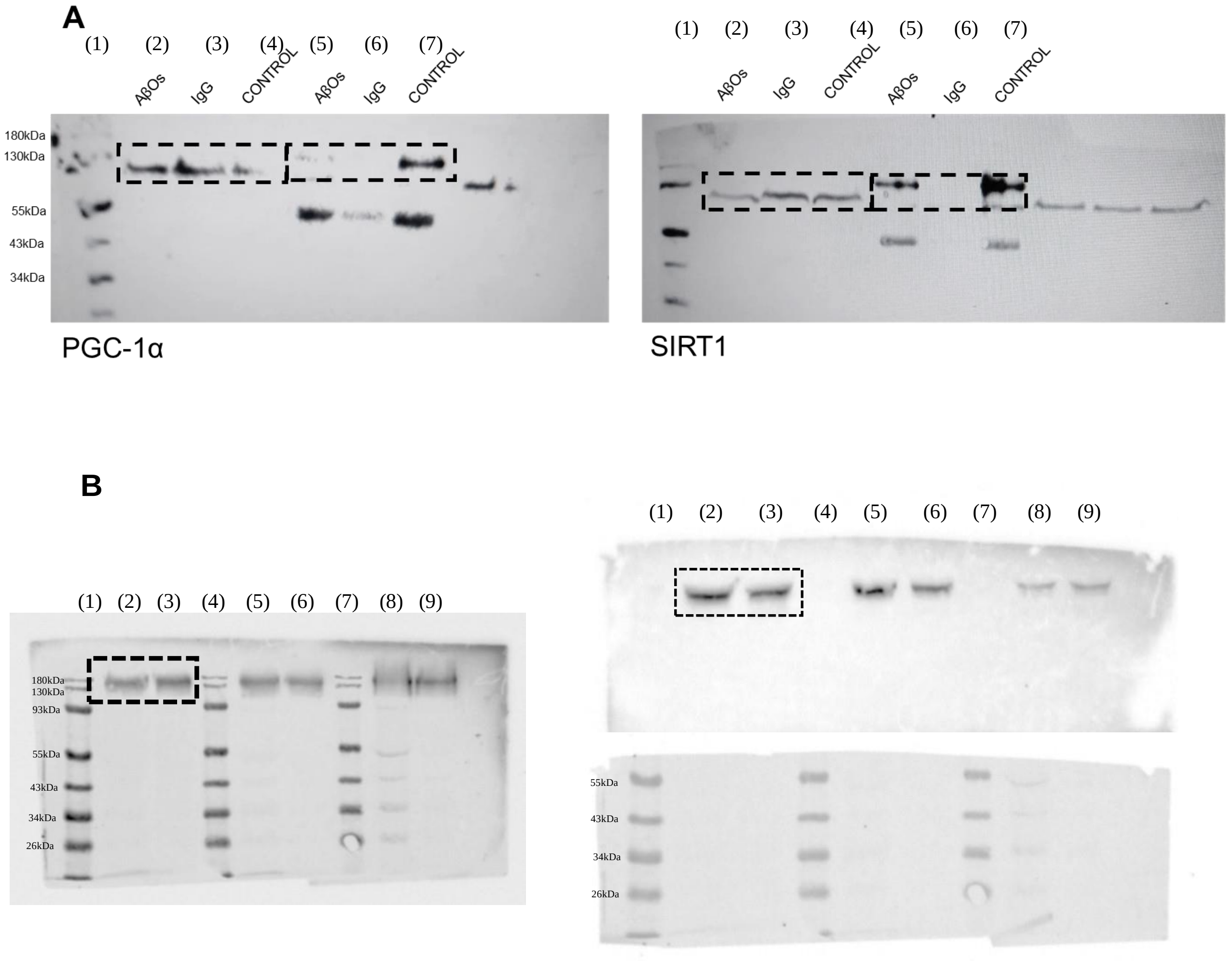

Supplementary Figure 9. A. Original gels for Figure 9A (shown in dotted line) for PGC-1α channel(100 kDa, left panel) SIRT1 (and MW channel(120 kDa, right panel)) in PC-12 cells after chronic treatment with SO-Aβ (0.5 µM; Iane 3). Lane 1 is Page ruler, lane 2 is chronic treatment with SO-Aβ, lane 3 is the IgG control and lane 4 is control condition. Co-immunoprecipitation are shown in lanes 5, where we can see chronic treatment with SO-Aβ, lane 6 corresponds a IgG control, and lane 7 corresponds to control condition. **B**. Original gels for Figure 9B (shown in dotted line) to SIRT1 (left panel) and beta actin (right panel). The lanes correspond to (1)Page ruler(2) Control (3) AβOs (24h) (4) Page ruler (5) Control (6) AβOs 1h (7) Page ruler (8) Control (9) AβOs 24h. We use beta actin as a loading control

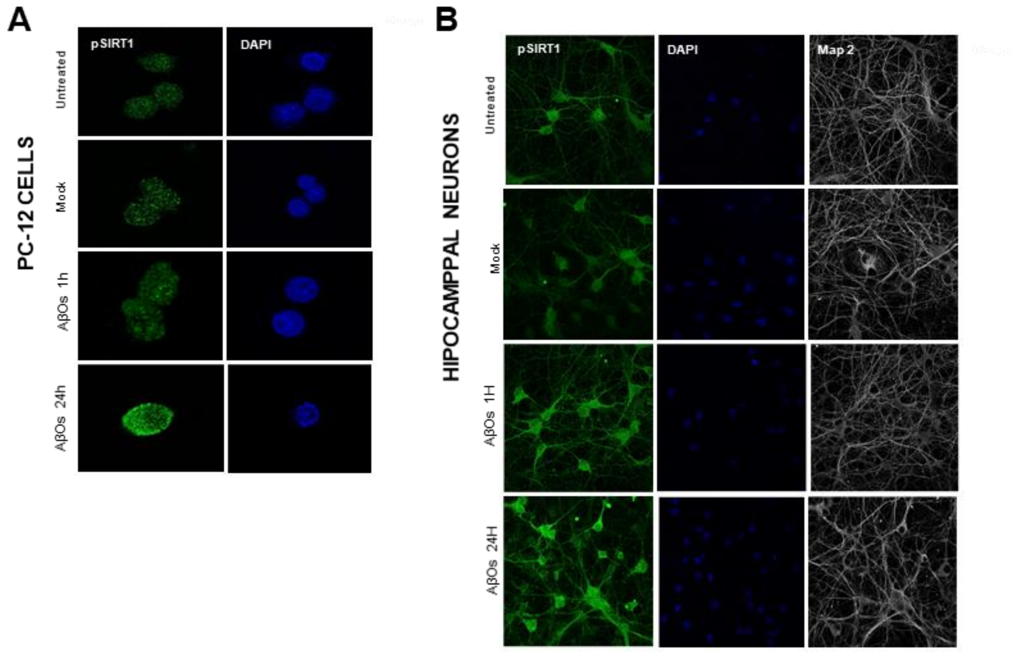

Supplementary Figure 10. Immunocytochemistry to evaluate p-SIRT1 distribution in PC12 cells and hippocampal neurons treated with AβOs. Representative confocal images of (A) PC12 cells and (B) hippocampal neurons showing the immunoreactivity of specific primary antibodies for ph-SIRT1 (green) and DAPI (blue) in control condition (untreated upper panel, mock oligomerization middle panel) and after AβOs treatment (0.5 µM) during different incubation times (1, 24h lower panels).

In review

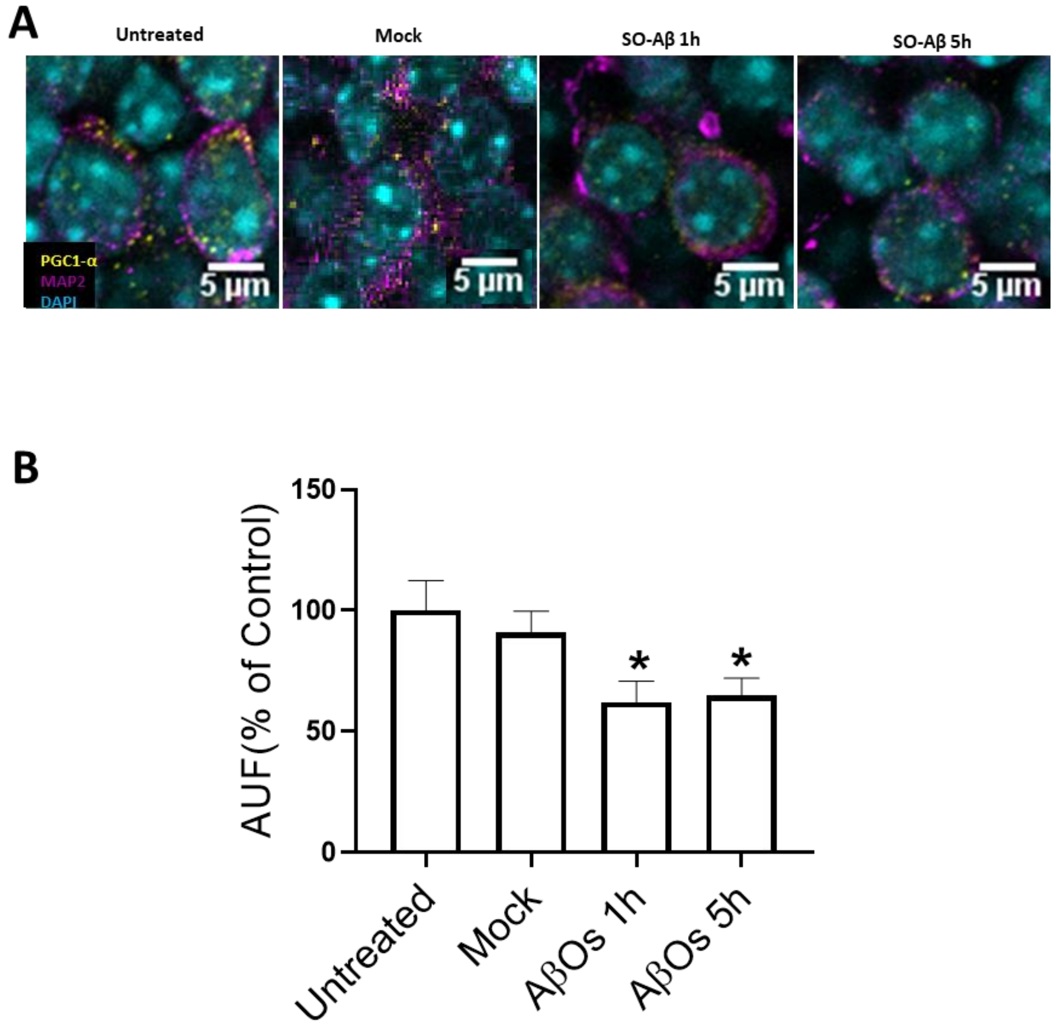

Supplementary Figure 11. Immunohistochemistry to show acute and sub-chronic effects of AβOs exposure on PGC1α in mice hippocampal slices. A) Maximum intensity projections of one confocal plane images showing neurons from the granular layer of the dentate gyrus in mice hippocampus. (C) Normalized quantification of the fluorescence intensity of PGC1α in control condition and after AβOs (0.5 μM) exposure during 1 or 5 hours.

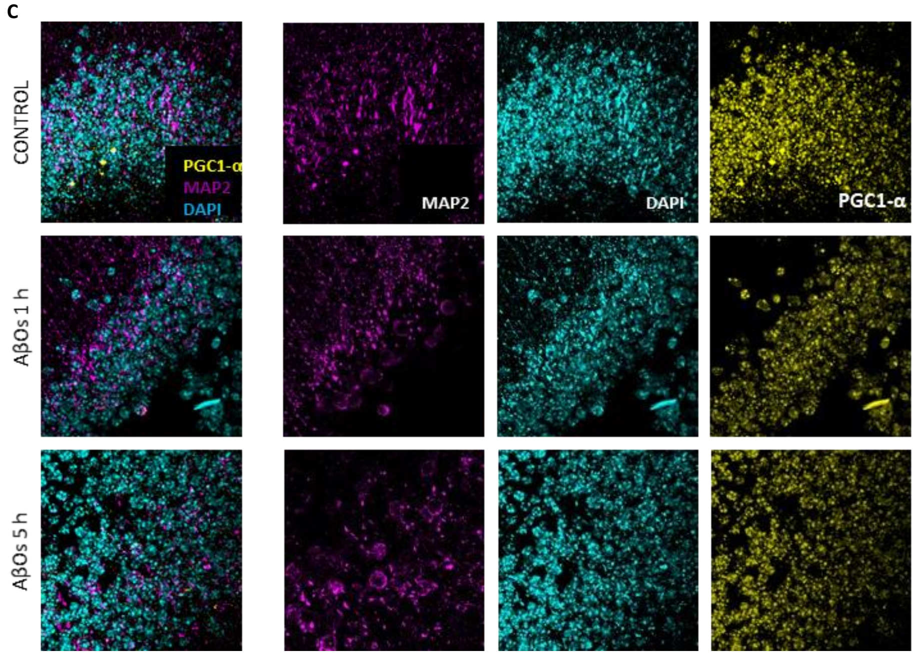

Supplementary Figure 12. Immunohistochemistry to show acute and sub-chronic effects of AβOs exposure on PGC1α in mice hippocampal slices. Representative confocal images hippocampal slices showing the immunoreactivity of specific primary antibodies Antibody immunoreactivity corresponds to: PGC1α (yellow, 1:400), MAP2 (violet, 1:400) and DAPI nuclear stain (cyan) to identify neurons. Data was analyzed using one way Anova with Kruskall-Wallis test and Dunn's Multiple Comparison test. (*p<0.05, control nroi: 462/nslices: 75, AβOs 1H nroi: 664/nslices: 37, AβOs 5H nroi: 977/nslices: 30).
